# Supplementary material for: Field Trial with Vaccine Candidates Against Bovine Tuberculosis Among Likely Infected Cattle in a Natural Transmission Setting
Source: Vaccines (Basel). 2024 Oct 17;12(10):1173. doi: 10.3390/vaccines12101173 (PMC11512252; doi:10.3390/vaccines12101173)
Supplement: Supplementary file 1 [file vaccines-12-01173-s001.zip › vaccines-3079652-supplementary.pdf]

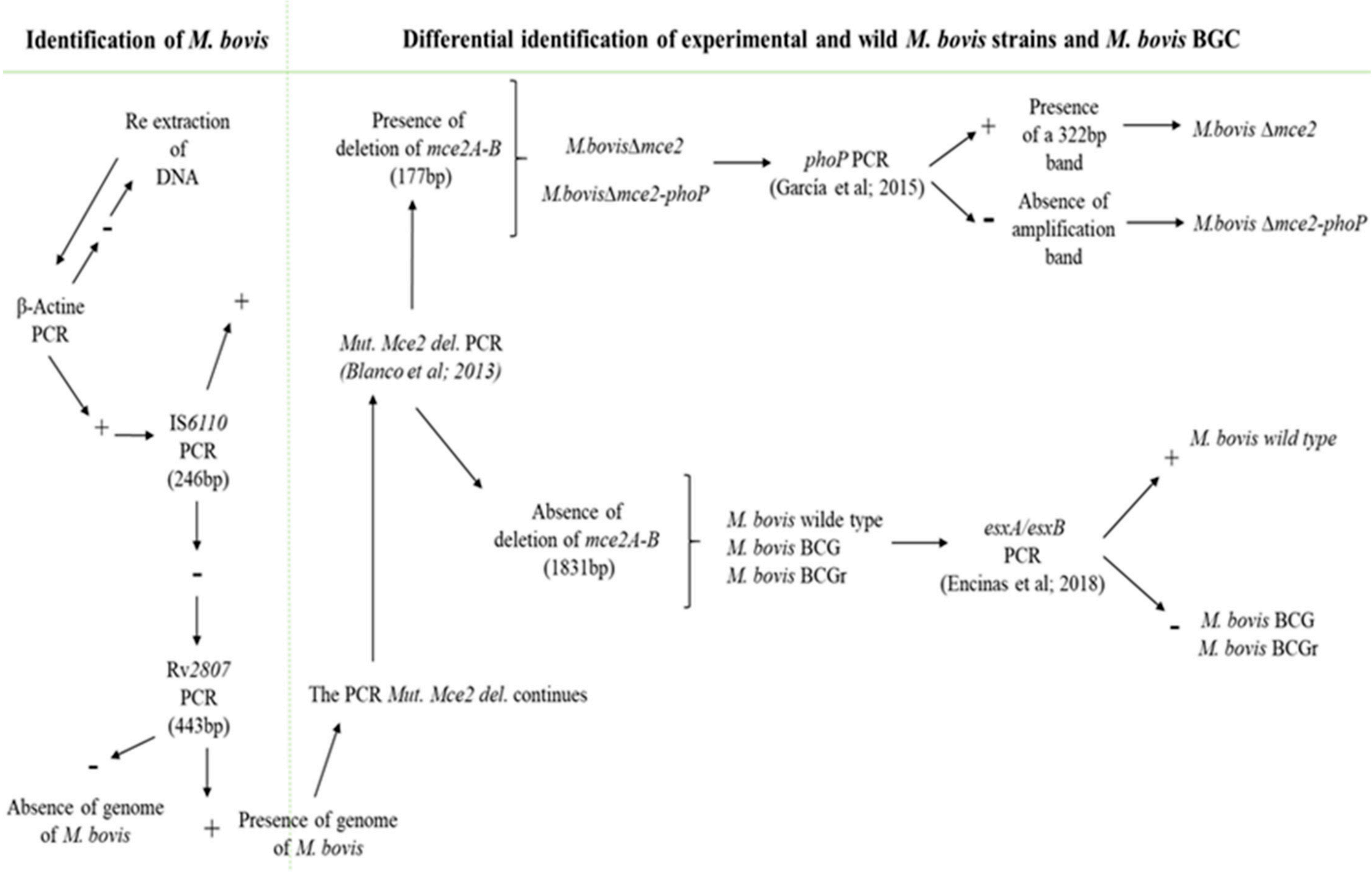

Supplemental material Figure S1. A schematic representation of the workflow used to perform the molecular identification by PCR of *M. bovis* strains included in the trial, based on the presence/absence of genomic sequences in the candidate vaccine strains, *M. bovis* BCG/BCGr and the wild-type *M. bovis* strains.
